# Atropia *Versus* Opium

**Published:** 1870-08

**Authors:** Theodore Griffin

**Affiliations:** 582 State Street, Chicago


ARTICLE XXI.

### ATROPIA *VERSUS* OPIUM.

By THEODORE GRIFFIN, M.D., 582 State Street, Chicago.

It is well established that the physiological effect of belladonna, and its alkaloid atropia, upon the human system is directly antagonistic, and opposite to that of opium and its alkaloid, morphia. We infer, therefore, that these drugs, if taken at the same time into the stomach of an individual, must neutralize the effects of each other; and this inference is well sustained by the abundant testimony which the experience of the past few years has given us. It is altogether probable that in belladonna we have found the most reliable antidote for poisoning by opium. The object of this communication is to add another link to the great chain of accumulating testimony in its favor.

In June last, I was called to see a person who was said to be dying. I hastily answered the summons, and found my patient rapidly sinking into a comatose condition; answered questions incoherently and in a whisper; cannot protrude his tongue; pupils much contracted; pulse 110 and weak; respirations six per minute; learned that the individual had recently taken 12 grains of opium. I at once ordered the administration of stimulants, friction of the extremities, sinipisms, with all auxilliary measures usually applied in similar conditions. He continued to sink, is now entirely unconscious; conjunctiva insensible to the touch; stertorous breathing; extremities cold and clammy; capillary congestion of the surface; respirations are now but three and four per minute, short and irregular, often no respiration during one-half minute, with three short, irregular ones during the following half minute; I believed the patient dying. Ordered, sulph. atropia, gr. j, aqua, ℥j, mix. Of this I injected at the point of insertion of the deltoid muscle, four minims, and in 15 minutes I repeated the injection; making the amount used 1-24 of a grain of the sulphate. I now discontinued all other means, and ordered the patient to be let alone. In half an hour the pupils began to dilate; in one and a half hours moderately dilated; in three hours pupils widely dilated; respirations five and six per minute, extremities warm, capillary congestions gone from the surface. At 12½ o'clock P.M., five hours from the period of injecting and two hours from the last recorded observations, conjunctiva responds to the touch, respirations nine per minute, pulse 100 and full, pupils well dilated, and all his symptoms much improved. At 3 P.M., nine hours from the time of the injection, awakes with a nervous start, and recognizes his friends; respirations 13 per minute, pulse 94; feels well.

I have no remarks to make as to the probable antidotal effects of the atropia in this case, it is too palpable to require discussion; from the extremest degree of prostration and impending death, the patient was raised to life.

